# Determinants for Autopsy after Unexplained Deaths Possibly Resulting from Infectious Causes, United States

**DOI:** 10.3201/eid1804.111311

**Published:** 2012-04

**Authors:** Lindy Liu, Laura S. Callinan, Robert C. Holman, Dianna M. Blau

**Affiliations:** Centers for Disease Control and Prevention, Atlanta, Georgia, USA

**Keywords:** coroners and medical examiners, autopsy, communicable diseases, epidemiology, United States, bacteria, viruses

## Abstract

Autopsy findings, clinical history, and diagnostic tools can aid surveillance and investigation of infectious diseases.

## Medscape CME Activity

Medscape, LLC is pleased to provide online continuing medical education (CME) for this journal article, allowing clinicians the opportunity to earn CME credit. 

This activity has been planned and implemented in accordance with the Essential Areas and policies of the Accreditation Council for Continuing Medical Education through the joint sponsorship of Medscape, LLC and Emerging Infectious Diseases. Medscape, LLC is accredited by the ACCME to provide continuing medical education for physicians.

Medscape, LLC designates this Journal-based CME activity for a maximum of 1 *AMA PRA Category 1 Credit(s)*^TM^. Physicians should claim only the credit commensurate with the extent of their participation in the activity.

All other clinicians completing this activity will be issued a certificate of participation. To participate in this journal CME activity: (1) review the learning objectives and author disclosures; (2) study the education content; (3) take the post-test with a 70% minimum passing score and complete the evaluation at www.medscape.org/journal/eid; (4) view/print certificate.

**Release date: March 15, 2012; Expiration date: March 15, 2013**

## Learning Objectives

Upon completion of this activity, participants will be able to:

- Assess characteristics of cases of unexplained deaths possibly resulting from infectious causes
- Distinguish the age group most likely to receive an autopsy after unexplained death
- Evaluate other variables associated with a higher likelihood of receiving an autopsy after unexplained death

## CME Editor

**Karen L. Foster, **Technical Writer/Editor, Emerging Infectious Diseases. *Disclosure: Karen L. Foster has disclosed no relevant financial relationships.*

## CME Author

**Charles P. Vega, MD, **Health Sciences Clinical Professor; Residency Director, Department of Family Medicine, University of California, Irvine. *Disclosure: Charles P. Vega, MD, has disclosed no relevant financial relationships.*

## Authors

Disclosures: Lindy Liu, MPH; Laura S. Callinan; Robert C. Holman, MS; and **Dianna M. Blau, DVM, PhD**, have disclosed no relevant financial relationships.
