# Supplementary material for: Determinants for Autopsy after Unexplained Deaths Possibly Resulting from Infectious Causes, United States
Source: Emerg Infect Dis. 2012 Apr;18(4):549–55. doi: 10.3201/eid1804.111311 (PMC3309683; doi:10.3201/eid1804.111311)
Supplement: Related texts

# Research article

**DOI:** 10.3201/eid1804.111311

## Abstract

We analyzed US multiple cause-of-death data for 2003–2006 for demographic and clinical determinants for autopsy in unexplained deaths possibly resulting from infectious causes. For 96,242 deaths, the definition for unexplained death was met and autopsy status was recorded. Most decedents were male, 40–49 years of age, and white. To identify factors associated with unexplained death, we used data from Arizona records. Multivariate analysis of Arizona records suggested that decedents of races other than white and black and decedents who had clinicopathologic syndromes in the cardiovascular, sepsis/shock, and multisyndrome categories recorded on the death certificate were least likely to have undergone autopsy; children with unexplained death were the most likely to have undergone autopsy. Improved understanding of unexplained deaths can provide opportunities for further studies, strengthen collaboration between investigators of unexplained deaths, and improve knowledge and awareness of infectious diseases of public health concern.

Many factors can influence the dynamics of pathogen ecology, increase the mobility of microbial agents, and elevate the risk for infectious disease posed to humans. Outbreaks and novel pathogens identified in recent decades are reminders that historical and newly recognized infectious diseases remain threats to the health of the global community (*1**–**3*). Unexplained deaths possibly resulting from infectious causes (unexplained deaths) also present public health challenges. Many fatal infectious etiologies are never identified because of inadequate testing or inherent difficulties of detecting certain pathogens (*4*).

An autopsy can verify an existing diagnosis or provide a diagnosis if one is not determined before death, which might facilitate provision of prophylaxis or treatment of contacts of decedents with communicable diseases. Autopsies also contribute to epidemiologic data, provide insights into disease pathogenesis, and create educational opportunities for physicians and medical students (*5*). Recent disease descriptions facilitated by autopsy findings include hantavirus pulmonary syndrome, West Nile virus, and severe acute respiratory syndrome (SARS) (*6**–**8*); pathogens for these diseases were recognized only after substantial numbers of illnesses and deaths. Although autopsies of persons who died of unexplained causes can help build public health capacity to respond to emerging infectious diseases, the declining rate of autopsies performed in US hospitals reduces the possibility of early detection of such diseases (*9*). Because most autopsies in the United States are now performed by medical examiners and coroners (*10*), medicolegal death investigation system–based surveillance for unexplained death can serve as a sentinel system to identify new agents, recognize unique characteristics of known pathogens, or detect acts of bioterrorism (*11*). Medical examiner and coroner systems contribute to national mortality data and autopsy-based information (*12*), and specimens collected at autopsy of persons whose deaths are unexplained could lead to diagnoses from advancements in diagnostic methods that have enabled identification and characterization of new infectious agents.

Although an earlier study measured unexplained deaths and critical illnesses (*4*), the demographic characteristics and clinicopathologic syndromes of persons whose deaths are unexplained who undergo autopsies have not been described. Understanding the types of persons who died of unexplained causes and who undergo autopsies might help identify specimens for diagnostic testing and improve epidemiologic and mortality data. We analyzed demographic characteristics and infectious disease–related syndromes associated with unexplained death in decedents for whom an autopsy was performed in the United States.

## Methods

### Data Source and Study Population

We obtained multiple cause-of-death data with autopsy status for 2003–2006 in the 50 states and the District of Columbia from the National Center for Health Statistics (NCHS), Centers for Disease Control and Prevention (*13*). Multiple cause-of-death data contain information from all death certificates for US residents, including demographic information and causes of death that have been translated to International Classification of Diseases, 10th Revision (ICD-10), codes (*14*).

On the basis of the previous definition of unexplained death (*15*) that was refined to use ICD-10 codes, we used 99 codes likely to represent deaths from unexplained infectious causes to select decedents for this study (Table A1). These codes aimed to capture deaths from infectious causes that lacked an identifiable etiologic agent or deaths with unknown causes. Unexplained deaths were defined as deaths of previously healthy US residents 1–49 years of age for whom the death certificate had >1 codes for unexplained infections. Decedents with unexplained infections for whom any of the ICD-10 codes listed in the Table 1 as an underlying cause of death were not considered previously healthy and were excluded from analysis. Decedents outside the age range also were excluded. We excluded infants (<1 year of age) to eliminate deaths attributed to congenital problems and persons >50 years of age because of the expected increased proportion of unexplained deaths from noninfectious causes (*15*). Analyses were restricted to unexplained deaths for which we could ascertain from the selected death certificate data whether an autopsy had been performed.

**Table 1:** Excluded ICD-10 codes and cause-of-death categories for unexplained deaths possibly resulting from infectious causes, United States, 2003–2006*

ICD-10 code	Cause-of-death category
B20-B24	HIV disease
C00-D48	Neoplasms
D73 (except D73.3)	Diseases of spleen
D80-D89	Certain disorders involving immune mechanism
E10-E14	Diabetes mellitus
F02.4	Dementia in HIV disease
R75	Inconclusive laboratory evidence of HIV
S00-T98	Injury, poisoning and certain consequences of external causes
V01-V99	Transport accidents
W00-X59	Other external causes of accidental injury
X60-X84	Intentional self-harm
X85-Y09	Assault
Y10-Y34	Event of undetermined intent
Y40-Y84	Complications of medical and surgical care

*ICD-10, International Classification of Diseases, 10th Revision.

### Study Measures and Statistical Analysis

Decedents were described by age, sex, and race and by the syndromic category recorded on the death certificate. Age was categorized as 1–17 years (children), 18–39 years, and 40–49 years. Race categories were white, black, and other, as recorded on death certificates and obtained from NCHS (*13*). Death certificates enabled reporting of >1 race, including any combination of white, black or African American, American Indian or Alaska Native, Asian, and Native Hawaiian or Other Pacific Islander, and decedents were imputed to a single race according to their combination of races, Hispanic origin, sex, and age indicated on the death certificate (*16*). On the basis of the selected ICD-10 codes, unexplained deaths were also grouped into 6 clinicopathologic syndromes: gastrointestinal, neurologic, respiratory, cardiovascular, sepsis/shock, and unknown/other (Table 2). Unexplained deaths for which ICD-10 codes were recorded as belonging to >2 syndromic categories were classified as multisyndrome.

**Table 2:** Syndromic classification of selected ICD-10 codes and cause of death for unexplained deaths possibly resulting from infectious causes, United States, 2003–2006*

Syndrome	ICD-10 codes
Gastrointestinal	A04.9, A05.9, A07.9, A08.4, A09, B82.0, B82.9, K29.7, K29.9, K51.9, K65.9, K85.9, R11, R85.5, R85.6, R85.7
Neurologic	A81.9, A83.9, A84.9, A85.2, A86, A87.9, A89, A92.9, A94, G00.9, G03.9, G04.9, G06.2, R29.8, R40.2, R83.5
Respiratory	J01.9, J02.9, J03.9, J06.9, J12.9, J15.9, J18.0, J18.1 J18.2, J18.8, J18.9, J20.9, J21.9, J22, R04.9, R84.5, R84.6, R84.7
Cardiovascular	D59.4, D59.9, D61.9, D64.9, D69.6, I01.9, I30.9, I33.9, I40.9, I42.8, I42.9, I51.4, I77.6, L95.9
Unknown/other	A28.9, A49.8, A49.9, A64, A68.9, A99, B09, B34.9, B49, B64, B83.9, B88.9, B89, B94.9, B99, D73.3, M60.0, N10.9, O98.9, P36.9, P37.9, P39.9, R50.9, R56.8, R59.9, R69, R89.5, R89.6, R89.7, R96.0, R96.1, R98, R99
Sepsis/shock	A41.9, R57.9

*ICD-10, International Classification of Diseases, 10th Revision.

We calculated odds ratios (ORs) with 95% CIs for selected characteristics by using logistic regression analysis. Characteristics considered univariately associated (p<0.1) with autopsy were further assessed through multivariate logistic regression models to determine which variables were independently associated with autopsy. We considered p<0.05 as significant. Because of the large sample size, statistical but not meaningful significance was found for most variables in the logistic regression model, including all unexplained deaths during 2003–2006 (data not shown). To further evaluate the variables, we created a multivariate logistic regression model using unexplained death data from Arizona for 2003–2006 (*17*). This subset of data was selected because of the minimal amount of missing autopsy data (0.2%) and the Unexplained Deaths Investigation Protocol, which identifies deaths that might be of public health concern, established by the Arizona Department of Health Services (*18*).

## Results

### United States

A total of 153,476 deaths were reported for persons 1–49 years of age for whom the selected ICD-10 codes (Table A1) were recorded in the multiple cause-of-death data for 2003–2006. Of these, 111,160 (72.4%) met the definition for unexplained death, and information on autopsy status was available for 96,242 (86.6%). Of decedents for whom autopsy status was known, 38,332 (39.8%) had undergone autopsy.

Of decedents for whom autopsy status was known, 59.5% were male (Table 3). Most decedents whose deaths were unexplained (55.1%) were 40–49 years of age; children accounted for 9.2%. Whites composed 71.7% of unexplained deaths, followed by blacks (24.6%) and others (3.7%). For most unexplained deaths, cause was coded as unknown/other syndrome (33.1%). Sepsis/shock accounted for 21.6%, and gastrointestinal and neurologic causes accounted for only 1.8% each.

**Table 3:** Characteristics of decedents 1–49 years of age and autopsies conducted for unexplained deaths possibly resulting from infectious causes, United States*

Characteristic	No. (%) decedents	No. (%) autopsies
Overall	96,242 (100)	38,332 (39.8)
Sex		
M	57,238 (59.5)	23,753 (41.5)
F	39,004 (40.5)	14,579 (37.4)
Race		
White	69,053 (71.7)	28,125 (40.7)
Black	23,657 (24.6)	9,006 (38.1)
Other†	3,532 (3.7)	1,201 (34.0)
Age group, y		
1–17	8,844 (9.2)	4,468 (50.5)
18–39	34,382 (35.7)	16,640 (48.4)
40–49	53,016 (55.1)	17,224 (32.5)
Syndrome		
Gastrointestinal	1,736 (1.8)	837 (48.2)
Neurologic	1,765 (1.8)	676 (38.3)
Respiratory	15,229 (15.8)	5,607 (36.8)
Cardiovascular	12,487 (13.0)	4,404 (35.3)
Sepsis/shock	20,762 (21.6)	3,298 (15.9)
Multisyndrome	12,371 (12.9)	2,688 (21.7)
Unknown/other	31,892 (33.1)	20,822 (65.3)

*Numbers reflect decedents for whom autopsy information was available; autopsy information was not available for 13.4% of the 111,160 persons who died of unexplained causes. †American Indian or Alaska Native, Asian Indian, Chinese, Filipino, Japanese, Korean, Vietnamese, Other Asian, Native Hawaiian, Guamanian or Chamorro, Samoan, other Pacific Islander, and other.

More male than female decedents underwent autopsies (41.5% vs. 37.4%) (Table 3). The highest percentage of autopsies was performed for white decedents (40.7%); autopsies were performed for 38.1% of black decedents and 34.0% of other decedents. Children whose deaths were unexplained underwent the highest percentage of autopsies (50.5%), followed by persons 18–39 years (48.4%) and 40–49 years of age (32.5%). The highest percentage of autopsies were performed on decedents whose cause of death was coded as unknown/other syndrome (65.3%); the lowest percentage of autopsies were performed on decedents whose deaths were coded as sepsis/shock syndrome (15.9%).

### Arizona

Of the 2,097 persons in Arizona who died from unexplained possibly infectious causes and for whom autopsy status was known, most (55.2%) were 40–49 years of age (Table 4). Whites composed 78.6% of such decedents, followed by others (14.4%) and blacks (7.1%). Most (33.9%) unexplained deaths resulted from other/unknown causes; unexplained deaths from gastrointestinal causes accounted for 1.2%.

**Table 4:** Association between having undergone autopsy and demographic characteristics of decedents 1–49 years of age and clinicopathologic syndrome for unexplained deaths possibly resulting from infectious causes, Arizona*

Characteristic	No. (%) decedents	No. (%) autopsies	Adjusted odds ratio (95% CI)
Total	2,097 (100.0)	696 (33.2)	
Race			
White	1,648 (78.6)	580 (35.2)	Reference
Black	148 (7.1)	53 (35.8)	1.0 (0.7–1.5)
Other†	301 (14.4)	63 (20.9)	0.5 (0.4–0.7)
Age group, y			
1–17	211 (10.1)	94 (44.5)	1.9 (1.4–2.6)
18–39	728 (34.7)	277 (38.0)	1.6 (1.3–2.0)
40–49	1,158 (55.2)	325 (28.1)	Reference
Syndrome			
Gastrointestinal	25 (1.2)	15 (60.0)	1.8 (0.8–4.1)
Neurologic	54 (2.6)	28 (51.9)	1.2 (0.7–2.1)
Respiratory	319 (15.2)	142 (44.5)	0.9 (0.7–1.2)
Cardiovascular	213 (10.2)	59 (27.7)	0.4 (0.3–0.6)
Sepsis/shock	428 (20.4)	62 (14.5)	0.2 (0.2–0.3)
Multisyndrome	348 (16.6)	56 (16.1)	0.2 (0.2–0.3)
Unknown/other	710 (33.9)	334 (47.0)	Reference

*By multivariate logistic regression analysis. Numbers reflect decedents for whom autopsy information was available; autopsy status was not available for 0.2% of the 2,102 persons who died of unexplained causes. Variables are independently associated with autopsy. †American Indian or Alaska Native, Asian Indian, Chinese, Filipino, Japanese, Korean, Vietnamese, Other Asian, Native Hawaiian, Guamanian or Chamorro, Samoan, Other Pacific Islander, and Other.

Percentages of decedents for whom an autopsy was performed were similar for whites (35.2%) and blacks (35.8%) (Table 4). The highest percentages of autopsies were performed on children whose deaths were unexplained (44.5%), followed by persons 18–39 years (38.0%) and 40–49 (28.1%) years of age. Of the 7 syndromic classifications, gastrointestinal cause of death accounted for the highest percentage of autopsies (60.0%) and sepsis/shock for the lowest percentage (14.5%).

Univariate analysis of data on persons who died from unexplained infectious causes in Arizona indicated that race, age group, and syndromic category, but not sex, were significantly associated with autopsy. Multivariate logistic regression analysis indicated that race, age group, and syndromic category remained independent predictors of autopsy. Persons of other races were less likely than white persons to undergo autopsy (OR 0.5, 95% CI 0.4–0.7) (Table 4). Children whose deaths were unexplained (OR 1.9, 95% CI 1.4–2.6) and persons 18–39 years of age (OR 1.6, 95% CI 1.3–2.0) were more likely to have undergone autopsy than were persons 40–49 years of age (Table 4). Persons with cardiovascular conditions, sepsis/shock, and multisyndrome conditions were less likely to have undergone autopsy than were persons with unknown/other unexplained deaths (Table 4).

## Discussion

Unlike other studies that have described and analyzed characteristics that influence autopsies overall (*19**,**20*), ours describes demographic characteristics and clinicopathologic syndromes associated with autopsy of persons who died of unexplained infectious causes in the United States. The overall percentage of autopsies performed on such decedents during 2003–2006 (39.8%) was higher than estimates of the proportion of overall autopsies in the United States (≈8.5%) (*21*). The higher percentage of autopsies for persons whose deaths were unexplained might reflect the frequent inclusion of complete autopsies in investigations of natural disease deaths by medical examiners and coroners (*22*).

Our finding that most characteristics in the multivariate regression analysis were highly significant when complete data for 2003–2006 were included in the analysis probably resulted from the large number of persons in the study whose deaths were unexplained. Unexplained deaths among persons with a history of fever have been reportable in Arizona since 2004, and medical examiners and health care providers are required to report unexplained infectious deaths to their local health departments (*18*). The Arizona Unexplained Deaths Investigation Protocol identifies appropriate specimens and clinical data needed for investigation, and the Arizona data might elucidate true demographic characteristics and syndromic trends of unexplained deaths in the United States. The analysis of data for Arizona decedents suggests that race, age, and clinicopathologic syndrome are potentially major factors for whether persons who died of unexplained infectious causes undergo autopsy.

Data on religious preferences are not collected on death certificates, but race might have been a proxy for cultural and religious preferences. Religious objections and lack of understanding about cultural or religious influences have been reported as reasons a family might not consent to an autopsy (*23**,**24*). For example, many American Indian tribes have traditions contrary to autopsy in which organ specimens are retained by medical examiners and pathologists (*25*). The observed lower odds for autopsy of decedents of other races possibly resulted from the larger American Indian population in Arizona (5%) than in the United States (1%) (*26*).

Results from the analysis of the Arizona subset suggest that children and young adults whose deaths resulted from unexplained possibly infectious causes are more likely than older adults to have undergone autopsies. Although some studies have suggested that children are more likely to undergo autopsies (*19**,**20*), the literature regarding the association between age and autopsy is limited, and findings have been inconclusive (*27**,**28*). Particularly when children die suddenly or unexpectedly, which is often from infectious causes (*29*), autopsies can contribute to families’ understanding of the circumstances of death or expand medical knowledge (*19**,**30*).

Persons whose unexplained deaths were coded as from cardiovascular, sepsis/shock, or multisyndrome causes were less likely than those whose deaths were coded as unknown/other to undergo autopsies. These results could reflect differences in the availability and resources of investigators of unexplained deaths from possibly infectious causes. Sepsis, in particular, remains perplexing and costly, and despite efforts to understand the systemic inflammation and multisystem organ failure characteristics of severe sepsis, the reason many of these patients die remains unknown (*31**,**32*). Furthermore, investigators of unexplained deaths or family members of decedents might have believed that additional studies, including autopsy, would not yield substantial findings. According to an opinion survey of pathology and medicine resident physicians, reasons families refuse autopsies included beliefs that the patient has suffered enough and that the autopsy would not be useful (*33*). Routine microscopic examination has been argued to not provide additional information in forensic pathology cases for which the cause and manner of death are apparent at the time of autopsy (*34*). However, the reduced likelihood of autopsy or further evaluation of these challenging unexplained deaths could also result in the failure to recognize infectious diseases. For example, Chong et al. illustrated the difficulty of differentiating an emerging disease (SARS) from other causes of sudden cardiovascular death at autopsy (*35*). Of the14 autopsies performed on persons with suspected or probable SARS, 8 confirmed SARS only on the basis of clinical history, histopathologic evaluation, and testing of autopsy specimens. Therefore, an autopsy should be pursued especially for those whose unexplained deaths were possibly of infectious causes.

Reasons for differences in likelihood of autopsy with respect to race, age, and clinicopathologic syndrome could be multifactorial, and results from our study are subject to limitations. The availability, training, and resources of investigators of unexplained natural deaths differ among institutions and jurisdictions and might account for differences in autopsy performance, testing capabilities, and reporting of autopsy data (*12**,**36*). Unfortunately, multiple cause-of-death data do not capture whether autopsies are performed by medical examiners or by hospital-based pathologists, and differences in autopsy rates between medicolegal death investigation systems and hospital-based pathologists in unexplained death remains unknown. Inaccuracy in death certification and reliance solely on ICD-10 classification for unexplained death also has limitations. Codes might be assigned by persons not directly familiar with decedents and who therefore might not be aware of known diagnoses. Death certificates might not have been amended when organism-specific etiologies (i.e., *Streptococcus pneumoniae*) were determined after broad ICD-10 codes (i.e., bacterial meningitis) were assigned. Results from our study also are limited by the restriction of analyses to unexplained deaths for which autopsy status is known and the large variation of autopsy data reported by states to NCHS. Of deaths that met the unexplained death definition, the percentage of missing autopsy status data by state ranged from 0 to 99% during 2003–2006. Additional data on autopsy status reported to NCHS could have more accurately described unexplained death.

Additional studies are needed to assess the similarities in demographic characteristics and clinicopathologic syndromes of persons who died of unexplained possibly infectious causes and characteristics found in autopsies overall. The statistically significant findings of such characteristics as age and race in this study could reflect general trends of autopsies performed and might not be unique to persons whose infectious deaths are unexplained. Furthermore, results from the analysis of Arizona data might not necessarily reflect unexplained deaths in other states or nationally.

Additional insight into persons who died of unexplained infectious causes and underwent autopsies might help pinpoint areas in which diagnostic capabilities or resources are needed (*15*) and provide opportunities for additional studies. Retrospective studies using postmortem specimens and improved diagnostic tools could benefit the broader community. Improved understanding by health departments and medical examiners of a specific type of unexplained death for which an autopsy is conducted could increase overall awareness of unexplained deaths from infectious causes; improve approaches in the collection of medical history and laboratory results in the forensic setting (*37*); and strengthen collaboration between health departments, clinicians, and medical examiners. Awareness of the types of unexplained death for which autopsies are less likely to be conducted is also imperative. Clinicians and pathologists challenged by cultural or religious restrictions can consider alternative methods for diagnosis such as taking biopsy samples (*38*), collecting appropriate antemortem specimens, or performing virtual autopsies (*39**,**40*). Retrospective studies evaluating perceptions by families, physicians, and medical examiners on autopsies of persons who died of unexplained infectious causes also might be helpful. Improving education about unexplained death and autopsy, identifying areas where diagnostic resources are needed, and maintaining cooperation between investigators should be considered. Autopsy findings, in conjunction with clinical history and diagnostic tools, can assist surveillance and investigations of infectious diseases of public health concern.

## Appendix

**Table A1:** Selected ICD-10 codes with corresponding cause of death for unexplained deaths possibly resulting from infectious causes, United States, 2003–2006*

ICD-10 code	Cause of death description
A04.9	Bacterial intestinal infection, unspecified
A05.9	Bacterial foodborne intoxication, unspecified
A07.9	Protozoal intestinal disease, unspecified
A08.4	Viral intestinal infection, unspecified
A09	Diarrhea and gastroenteritis of presumed infectious origin
A28.9	Zoonotic bacterial disease, unspecified
A41.9	Septicemia, unspecified
A49.8	Other bacterial infections of unspecified site
A49.9	Bacterial infection, unspecified
A64	Unspecified sexually transmitted disease
A68.9	Relapsing fever, unspecified
A81.9	Atypical virus infection of central nervous system, unspecified
A83.9	Mosquito-borne viral encephalitis, unspecified
A84.9	Tick-borne viral encephalitis, unspecified
A85.2	Arthropod-borne viral encephalitis, unspecified
A86	Unspecified viral encephalitis
A87.9	Viral meningitis, unspecified
A89	Unspecified viral infection of central nervous system
A92.9	Mosquito-borne viral fever, unspecified
A94	Unspecified arthropod-borne viral fever
A99	Unspecified viral hemorrhagic fever
B09	Unspecified viral infection characterized by skin and mucous membrane lesions
B34.9	Viral infection, unspecified
B49	Unspecified mycosis
B64	Unspecified protozoal disease
B82.0	Intestinal helminthiasis, unspecified
B82.9	Intestinal parasitism, unspecified
B83.9	Helminthiasis, unspecified
B88.9	Infestation, unspecified
B89	Unspecified parasitic disease
B94.9	Sequelae of unspecified infectious or parasitic disease
B99	Other and unspecified infectious diseases
D59.4	Other nonautoimmune hemolytic anemias
D59.9	Acquired hemolytic anemia, unspecified
D61.9	Aplastic anemia, unspecified
D64.9	Anemia, unspecified
D69.6	Thrombocytopenia, unspecified
D73.3	Abscess of spleen
G00.9	Bacterial meningitis, unspecified
G03.9	Meningitis, unspecified
G04.9	Encephalitis, myelitis and encephalomyelitis, unspecified
G06.2	Extradural and subdural abscess, unspecified
I01.9	Acute rheumatic heart disease, unspecified
I30.9	Acute pericarditis, unspecified
I33.9	Acute endocarditis, unspecified
I40.9	Acute myocarditis, unspecified
I42.8	Other cardiomyopathies
I42.9	Cardiomyopathy, unspecified
I51.4	Myocarditis, unspecified
I77.6	Arteritis, unspecified
J01.9	Acute sinusitis, unspecified
J02.9	Acute pharyngitis, unspecified
J03.9	Acute tonsillitis, unspecified
J06.9	Acute upper respiratory infection, unspecified
J12.9	Viral pneumonia, unspecified
J15.9	Bacterial pneumonia, unspecified
J18.0	Bronchopneumonia, unspecified
J18.1	Lobar pneumonia, unspecified
J18.2	Hypostatic pneumonia, unspecified
J18.8	Other pneumonia, organism unspecified
J18.9	Pneumonia, unspecified
J20.9	Acute bronchitis, unspecified
J21.9	Acute bronchiolitis, unspecified
J22	Unspecified acute lower respiratory infection
K29.7	Gastritis, unspecified
K29.9	Gastroduodenitis, unspecified
K51.9	Ulcerative colitis, unspecified
K65.9	Peritonitis, unspecified
K85.9	Acute pancreatitis, unspecified
L95.9	Vasculitis limited to skin, unspecified
M60.0	Infective myositis
N10.9	Acute tubulo-interstitial nephritis
O98.9	Unspecified maternal infectious or parasitic disease complicating pregnancy, childbirth and the puerperium
P36.9	Bacterial sepsis of newborn, unspecified
P37.9	Congenital infectious and parasitic disease, unspecified
P39.9	Infectious specific to the perinatal period, unspecified
R04.9	Hemorrhage from respiratory passages, unspecified
R11	Nausea and vomiting
R29.8	Other and unspecified symptoms and signs involving the nervous and musculoskeletal systems
R40.2	Coma, unspecified
R50.9	Fever, unspecified
R56.8	Other and unspecified convulsions
R57.9	Shock, unspecified
R59.9	Enlarged lymph nodes, unspecified
R69	Unknown and unspecified causes of morbidity
R83	Abnormal findings in cerebrospinal fluid
R83.5	Abnormal microbiological findings, positive culture findings
R84	Abnormal findings in specimens from respiratory organs and thorax
R84.5	Abnormal microbiological findings, positive culture findings
R84.6	Abnormal cytological findings, Abnormal Papanicolaou smear
R84.7	Abnormal histological findings
R85	Abnormal findings in specimens from digestive organs and abdominal cavity
R85.5	Abnormal microbiological findings, positive culture findings
R85.6	Abnormal cytological findings, abnormal Papanicolaou smear
R85.7	Abnormal histological findings
R89	Abnormal findings in specimens from other organs, systems, and tissues
R89.5	Abnormal microbiological findings, positive culture findings
R89.6	Abnormal cytological findings, abnormal Papanicolaou smear
R89.7	Abnormal histological findings
R96.0	Instantaneous death
R96.1	Death occurring less than 24 h from onset of symptoms, not otherwise explained
R98	Unattended death
R99	Other ill-defined and unspecified causes of mortality

*ICD-10, International Classification of Diseases, 10th Revision.

## References

[1] Campbell GL, Hughes JM. Plague in India: a new warning from an old nemesis. Ann Intern Med. 1995;122:151–3.799299210.7326/0003-4819-122-2-199501150-00014

[2] Fischer SA, Graham MB, Kuehnert MJ, Kotton CN, Srinivasan A, Marty FM, Transmission of lymphocytic choriomeningitis virus by organ transplantation. N Engl J Med. 2006;354:2235–49. 10.1056/NEJMoa05324016723615

[3] Novel Swine-Origin Influenza A (H1N1) Virus Investigation Team; Dawood FS, Jain S, Finelli L, Shaw MW, Lindstrom S, Emergence of a novel swine-origin influenza A (H1N1) virus in humans. N Engl J Med. 2009;360:2605–15. 10.1056/NEJMoa090381019423869

[4] Hajjeh RA, Relman D, Cieslak PR, Sofair AN, Passaro D, Flood J, Surveillance for unexplained deaths and critical illnesses due to possibly infectious causes, United States, 1995–1998. Emerg Infect Dis. 2002;8:145–53. 10.3201/eid0802.01016511897065PMC2732455

[5] McPhee SJ. Maximizing the benefits of autopsy for clinicians and families. What needs to be done. Arch Pathol Lab Med. 1996;120:743–8.8718899

[6] Zaki SR, Greer PW, Coffield LM, Goldsmith CS, Nolte KB, Foucar K, Hantavirus pulmonary syndrome. Pathogenesis of an emerging infectious disease. Am J Pathol. 1995;146:552–79.7887439PMC1869168

[7] Shieh WJ, Guarner J, Layton M, Fine A, Miller J, Nash D, The role of pathology in an investigation of an outbreak of West Nile encephalitis in New York, 1999. Emerg Infect Dis. 2000;6:370–2. 10.3201/eid0604.00040710905969PMC2640902

[8] Nicholls JM, Poon LL, Lee KC, Ng WF, Lai ST, Leung CY, Lung pathology of fatal severe acute respiratory syndrome. Lancet. 2003;361:1773–8. 10.1016/S0140-6736(03)13413-712781536PMC7112492

[9] Dalen JE. The moribund autopsy. DNR or CPR? Arch Intern Med. 1997;157:1633. 10.1001/archinte.1997.004403600190019250221

[10] Hanzlick R. Medical examiners, coroners, and public health: a review and update. Arch Pathol Lab Med. 2006;130:1274–82.1694851110.5858/2006-130-1274-MECAPH

[11] Nolte KB, Fischer M, Reagan S, Lynfield R. Guidelines to implement medical examiner/coroner-based surveillance for fatal infectious diseases and bioterrorism (“Med-X”). Am J Forensic Med Pathol. 2010;31:308–12. 10.1097/PAF.0b013e3181c187b520683243

[12] Hanzlick R. The conversion of coroner systems to medical examiner systems in the United States: a lull in the action. Am J Forensic Med Pathol. 2007;28:279–83. 10.1097/PAF.0b013e31815b4d5a18043011

[13] National Center for Health Statistics. Vital statistics mortality data, multiple cause detail, 2003–2006. Public use data tape contents and documentation package. Hyattsville (MD): The Center; 2008.

[14] Centers for Disease Control and Prevention. Multiple cause of death 1999−2006 [cited 2011 Nov 15]. http://wonder.cdc.gov/wonder/help/mcd.html

[15] Perkins BA, Flood JM, Danila R, Holman RC, Reingold AL, Klug LA, Unexplained deaths due to possibly infectious causes in the United States: defining the problem and designing surveillance and laboratory approaches. The Unexplained Deaths Working Group. Emerg Infect Dis. 1996;2:47–53. 10.3201/eid0201.9601068903196PMC2639807

[16] Ingram DD, Parker JD, Schenker N, Weed JA, Hamilton B, Arias E, United States Census 2000 population with bridged race categories. Vital Health Stat 2. 2003;(135):1–55.14556588

[17] Kleinbaum D, Klein M. Logisitc regression: a self-learning text. 2nd ed. New York: Springer-Verlag; 2002.

[18] Arizona Department of Health Services, Infectious Disease Epidemiology Program. Unexplained deaths with history of fever (UNEX) [cited 2011 Nov 15]. http://www.azdhs.gov/phs/oids/epi/unex/index.htm

[19] Sinard JH. Factors affecting autopsy rates, autopsy request rates, and autopsy findings at a large academic medical center. Exp Mol Pathol. 2001;70:333–43. 10.1006/exmp.2001.237111418012

[20] Andrews-Joseph A, Bourgeois SS, Ratard RC. Louisiana autopsy patterns 1999–2006. J La State Med Soc. 2009;161(2):97, 99–102. 19489390

[21] Hoyert DL. The changing profile of autopsied deaths in the United States, 1972–2007. NCHS data brief. No. 67, August 2011. Hyattsville (MD): US Department of Health and Human Services, Centers for Disease Control and Prevention, National Center for Health Statistics; 2011 [cited 2011 Nov 15]. http://www.cdc.gov/nchs/data/databriefs/db67.htm

[22] Wolfe MI, Nolte KB, Yoon SS. Fatal infectious disease surveillance in a medical examiner database. Emerg Infect Dis. 2004;10:48–53.1507859610.3201/eid1001.020764PMC3322763

[23] Geller SA. Religious attitudes and the autopsy. Arch Pathol Lab Med. 1984;108:494–6.6547302

[24] Perkins HS, Supik JD, Hazuda HP. Autopsy decisions: the possibility of conflicting cultural attitudes. J Clin Ethics. 1993;4:145–54.8334278

[25] Krinsky CS, Lathrop SL, Reichard RR. A policy for the retention and extended examination of organs at autopsy. J Forensic Sci. 2010;55:418–22. 10.1111/j.1556-4029.2009.01271.x20102456

[26] US Census Bureau. State and county quick facts: Arizona [cited 2011 Nov 15]. http://quickfacts.census.gov/qfd/states/04000.html

[27] Whitehouse SR, Kissoon N, Singh N, Warren D. The utility of autopsies in a pediatric emergency department. Pediatr Emerg Care. 1994;10:72–5. 10.1097/00006565-199404000-000028029113

[28] Ahronheim JC, Bernholc AS, Clark WD. Age trends in autopsy rates. Striking decline in late life. JAMA. 1983;250:1182–6. 10.1001/jama.1983.033400900380266876357

[29] Taggart MW, Craver R. Causes of death, determined by autopsy, in previously healthy (or near-healthy) children presenting to a children’s hospital. Arch Pathol Lab Med. 2006;130:1780–5.1714995010.5858/2006-130-1780-CODDBA

[30] Beckwith JB. The value of the pediatric postmortem examination. Pediatr Clin North Am. 1989;36:29–36.291354410.1016/s0031-3955(16)36614-7

[31] Angus DC, Linde-Zwirble WT, Lidicker J, Clermont G, Carcillo J, Pinsky MR. Epidemiology of severe sepsis in the United States: analysis of incidence, outcome, and associated costs of care. Crit Care Med. 2001;29:1303–10. 10.1097/00003246-200107000-0000211445675

[32] Torgersen C, Moser P, Luckner G, Mayr V, Jochberger S, Hasibeder WR, Macroscopic postmortem findings in 235 surgical intensive care patients with sepsis. Anesth Analg. 2009;108:1841–7. 10.1213/ane.0b013e318195e11d19448210

[33] Hull MJ, Nazarian RM, Wheeler AE, Black-Schaffer WS, Mark EJ. Resident physician opinions on autopsy importance and procurement. Hum Pathol. 2007;38:342–50. 10.1016/j.humpath.2006.08.01117134740

[34] Molina DK, Wood LE, Frost RE. Is routine histopathologic examination beneficial in all medicolegal autopsies? Am J Forensic Med Pathol. 2007;28:1–3. 10.1097/01.paf.0000257388.83605.0a17325455

[35] Chong PY, Chui P, Ling AE, Franks TJ, Tai DY, Leo YS, Analysis of deaths during the severe acute respiratory syndrome (SARS) epidemic in Singapore: challenges in determining a SARS diagnosis. Arch Pathol Lab Med. 2004;128:195–204.1473628310.5858/2004-128-195-AODDTS

[36] Wilson ML, Gradus S, Zimmerman SJ. The role of local public health laboratories. Public Health Rep. 2010;125(Suppl 2):118–22.2051845310.1177/00333549101250S215PMC2846811

[37] Christiansen LR, Collins KA. Natural death in the forensic setting: a study and approach to the autopsy. Am J Forensic Med Pathol. 2007;28:20–3. 10.1097/01.paf.0000233553.19938.a017325458

[38] Huston BM, Malouf NN, Azar HA. Percutaneous needle autopsy sampling. Mod Pathol. 1996;9:1101–7.8972468

[39] Thali MJ, Yen K, Schweitzer W, Vock P, Boesch C, Ozdoba C, Virtopsy, a new imaging horizon in forensic pathology: virtual autopsy by postmortem multislice computed tomography (MSCT) and magnetic resonance imaging (MRI)—a feasibility study. J Forensic Sci. 2003;48:386–403.12665000

[40] Thali MJ, Dirnhofer R, Becker R, Oliver W, Potter K. Is ‘virtual histology’ the next step after the ‘virtual autopsy’? Magnetic resonance microscopy in forensic medicine. Magn Reson Imaging. 2004;22:1131–8. 10.1016/j.mri.2004.08.01915528000

# Research article: Determinants for Autopsy after Unexplained Deaths Possibly Resulting from Infectious Causes, United States

**DOI:** 10.3201/eid1804.111311

**Authors:** Lindy Liu, Laura L. Sinden, Robert C. Holman, Dianna M. Blau

**Affiliations:** Centers for Disease Control and Prevention, Atlanta, Georgia, USA

## Medscape CME Quiz

## Earning CME Credit

To obtain credit, you should first read the journal article. After reading the article, you should be able to answer the following, related, multiple-choice questions. To complete the questions (with a minimum 70% passing score) and earn continuing medical education (CME) credit, please go to www.medscape.org/journal/eid. Credit cannot be obtained for tests completed on paper, although you may use the worksheet below to keep a record of your answers. You must be a registered user on Medscape.org. If you are not registered on Medscape.org, please click on the New Users: Free Registration link on the left hand side of the website to register. Only one answer is correct for each question. Once you successfully answer all post-test questions you will be able to view and/or print your certificate. For questions regarding the content of this activity, contact the accredited provider, CME@medscape.net. For technical assistance, contact CME@webmd.net. American Medical Association’s Physician’s Recognition Award (AMA PRA) credits are accepted in the US as evidence of participation in CME activities. For further information on this award, please refer to http://www.ama-assn.org/ama/pub/category/2922.html. The AMA has determined that physicians not licensed in the US who participate in this CME activity are eligible for *AMA PRA Category 1 Credits*™. Through agreements that the AMA has made with agencies in some countries, AMA PRA credit may be acceptable as evidence of participation in CME activities. If you are not licensed in the US, please complete the questions online, print the certificate and present it to your national medical association for review.

## Article Title: Determinants for Autopsy after Unexplained Deaths Possibly Resulting from Infectious Causes, United States

## CME Questions

**1. You are part of a county task force charged with developing means to monitor emerging infections, and you are preparing for a discussion of unexplained deaths possibly resulting from infectious causes (UDPIC).**

**In the current study, what was a significant characteristic of cases of UDPIC?**

A. Most cases occurred among male patients

B. Most cases occurred among children

C. Most cases occurred among persons of black race

D. Most cases occurred among persons of races other than black or white

**2. Which of the following was the most common category of UDPIC from the national sample in the current study?**

A. Sepsis/shock

B. Gastrointestinal disease

C. Neurologic disease

D. Unknown/other syndrome

**3. Which of the following age groups was most likely to have received an autopsy in the current study of UDPIC?**

A. Children and adolescents under age 18 years

B. Adults age 18–39 years

C. Adults age 40–49 years 

D. Adults age 50 or older

**4. What other characteristic was most associated with receiving an autopsy in the current study?**

A. UDPIC due to sepsis/shock

B. Living in an urban center

C. White race

D. Race other than white or black

## Activity Evaluation

**Table:** 

1. The activity supported the learning objectives.				
Strongly Disagree				Strongly Agree
1	2	3	4	5
2. The material was organized clearly for learning to occur.				
Strongly Disagree				Strongly Agree
1	2	3	4	5
3. The content learned from this activity will impact my practice.				
Strongly Disagree				Strongly Agree
1	2	3	4	5
4. The activity was presented objectively and free of commercial bias.				
Strongly Disagree				Strongly Agree
1	2	3	4	5
